# Computation of domination degree-based topological indices using python and QSPR analysis of physicochemical and ADMET properties for heart disease drugs

**DOI:** 10.3389/fchem.2025.1536199

**Published:** 2025-03-12

**Authors:** Geethu Kuriachan, A. Parthiban

**Affiliations:** Department of Mathematics, School of Advanced Sciences, Vellore Institute of Technology, Vellore, Tamil Nadu, India

**Keywords:** domination degree, minimal dominating sets, domination topological indices, ADMET properties, QSPR study

## Abstract

Heart disease is a leading cause of death worldwide, highlighting the need for effective treatments for hypertension, arrhythmias, and high cholesterol. This study applies chemical graph theory to analyze the properties of seventeen heart disease drugs by evaluating minimal dominating sets and counting node appearances in these sets. Using Python, six domination degree-based topological indices from the $\phi_{d}$ -polynomial are computed. Regression analysis, including curvilinear and multilinear models, identified correlations between these indices and the physicochemical and ADMET properties of these drugs. QSPR models are developed to assess the ability of these indices to predict key properties, offering insights into their effectiveness for drug design.

## 1 Introduction

Heart disease drugs, also known as cardiovascular drugs, are essential in treating conditions affecting the heart and blood vessels, including hypertension, coronary artery disease, heart failure, and arrhythmias. Beta blockers such as atenolol, metoprolol, esmolol, nadolol, propranolol, and timolol reduce heart rate and blood pressure, making them effective for hypertension, angina, and heart attack prevention. Diuretics like amiloride, chlortalidone, hydrochlorothiazide, furosemide, triamterene, and bumetanide manage fluid retention and high blood pressure by promoting the excretion of excess salt and water. Other drugs, including methyldopa, minoxidil, clonidine, and enalapril, lower blood pressure through various mechanisms as beta-blockers and diuretics are also anti-hypertensives. Antianginal drugs like nitroglycerin alleviate chest pain by increasing blood flow to the heart, while anticoagulants and antiplatelet drugs such as warfarin and clopidogrel prevent blood clots. Antiarrhythmics like sotalol regulate abnormal heart rhythms. These drugs play a vital role in managing heart disease by addressing both symptoms and underlying causes.

The selection of these heart disease drugs for analysis reflects their importance in treating conditions like hypertension, arrhythmias, and thrombosis, as well as their diverse pharmacological mechanisms. These drugs face unique ADMET (Absorption, Distribution, Metabolism, Excretion, and Toxicity) challenges, such as variable bioavailability due to solubility and gastrointestinal metabolism, seen with nitroglycerin and warfarin (Hardman and Limbird, 2001). Lipophilicity influences their distribution, affecting tissue targeting and blood-brain barrier permeability, particularly in drugs like propranolol and clonidine (Lipinski et al., 1997). Hepatic metabolism, often mediated by cytochrome P450 enzymes, poses risks of drug-drug interactions, as observed with clopidogrel and metoprolol. Renal clearance is a key factor for diuretics like furosemide and hydrochlorothiazide, while toxicity risks, such as arrhythmias with sotalol or severe hypotension with minoxidil, highlight safety challenges (Zanger and Schwab, 2013). Analyzing these drugs provides valuable insights into their molecular properties and enhances ADMET predictions through Quantitative Structure-Property Relationship (QSPR) modeling.

Topological indices (TIs) or molecular descriptors are mathematical representations of a compound’s molecular structure, where atoms are treated as vertices (nodes) and chemical bonds as edges (lines) in a graph. These indices capture key structural features such as atom connectivity, branching, and the overall shape of the molecule. TIs play a crucial role in various fields, particularly in QSPR and Quantitative Structure-Activity Relationship (QSAR) analyses, which predict the physicochemical and biological properties of molecules based on their structural characteristics. This provides valuable insights in drug design, environmental chemistry, and material science. The development of TIs began in the 1940s with early work in chemical graph theory. Wiener introduced molecular graphs in 1947 and proposed the Wiener index, a distance-based descriptor based on the sum of distances between all pairs of vertices in a molecule’s graph (Wiener, 1947). This laid the foundation for advancements in molecular property prediction. In 1975, Randic introduced the Randic index, which measures the balance of connectivity between atoms and predicts various physicochemical properties, including boiling point, solubility, and molecular weight (Randic, 1975). The development of computational tools and these indices enabled systematic exploration of molecular structure-property relationships, with QSPR/QSAR becoming a powerful tool in cheminformatics by the 1990s for modeling properties such as toxicity, bioactivity, and chemical reactivity. The development of more advanced indices, such as geometric and topological polar surface area, molecular volume, and electronic descriptors, further expanded the scope of QSPR/QSAR models. Using TIs and other molecular descriptors, such as electronic and geometric features, QSPR analyses develop statistical models that correlate chemical structure with desired properties. There is a potential to use QSPR indices to aid in accelerating drug discovery and material design.

Extensive research has been conducted to explore the applications of TIs in QSPR analyses, particularly in understanding the physicochemical and ADMET properties of chemical structures. Shanmukha et al. utilized degree-based TIs for anticancer drugs, combined with QSPR analysis, to establish correlations with various physicochemical properties (Shanmukha et al., 2020). Tamilarasi and Balamurugan extended QSPR studies to anti-COVID drugs targeting the Omicron variant, incorporating degree-based TIs for ADMET evaluations (Tamilarasi and Balamurugan, 2022). In a recent study (Tamilarasi and Balamurugan, 2024), new reverse sum Revan indices were introduced for analyzing the physicochemical and pharmacokinetic properties of anti-filovirus drugs, highlighting the adaptability of TIs in predicting the properties of diverse drug categories. Similarly, Mahboob et al. explored molecular descriptors in QSPR analysis for kidney cancer therapeutics (Mahboob et al., 2024b) and applied linear regression models to analyze anti-hepatitis drugs (Mahboob et al., 2024a). Muhammad Shoaib et al. conducted a QSPR analysis of Alzheimer’s compounds using TIs and regression models to effectively predict key properties (Sardar and Hakami, 2024). Chaluvaraju and T. (2024) performed a QSPR analysis of the generalized irregular neighborhood valency descriptor for some basic polycyclic aromatic hydrocarbons, while in another study (Asha et al., 2022), they investigated different types of augmented Zagreb indices for selected chemical drugs, developing a QSPR model. Additional advancements in this area can be found in the works (Sardar et al., 2024; Sardar et al., 2020; Sardar et al., 2025; Sardar and Xu, 2024). Collectively, these studies emphasize the critical role of TIs in enhancing the predictive power and reliability of QSPR models, facilitating advancements in drug design and the evaluation of therapeutic compounds.

Among the prominent contemporary TIs, domination topological indices (DTIs), introduced by Ahmed A. M. Hanan et al. (2021), are noteworthy for their innovative integration of two fundamental concepts in graph theory: topological indices and domination. These indices are intrinsically linked to the minimal dominating sets of a chemical graph, providing a unique perspective on the structural properties of molecular graphs. Although a few studies have focused on domination topological indices (Wazzan and Ahmed, 2024; Shashidhara R. et al., 2023; Shashidhara A. A. et al., 2023; Javaraju et al., 2021), research in this area remains limited. Furthermore, while several studies have focused on the QSPR analysis of the physicochemical properties of heart disease drugs (Arockiaraj et al., 2024; Hasani and Ghods, 2023; Hakeem et al., 2024), no research has been conducted on their ADMET properties. Therefore, this study is unique and can assist chemists in predicting the ADMET properties of these drugs.

There are eight sections in this article. Section 2 introduces the fundamental formulae used in the calculations. The methodology and working approach are detailed in Section 3. Section 4 presents the study’s primary findings, including the evaluation of six domination topological indices for seventeen heart disease drugs. In Section 5, inverse linear, quadratic, and cubic regression models are developed, and the correlations between the indices and properties are analyzed. This section also provides a detailed discussion on the significance of DTIs and compares the results comprehensively. Section 6 covers multilinear regression analysis, offering further insights into the relationships between the indices and drug properties. Section 7 provides a thorough analysis of the study’s findings, highlighting their potential contributions to existing literature, as well as discussing the broader implications and limitations of the research. The article concludes with a summary in Section 8, followed by a list of relevant references.

## 2 Basic definitions

Given a connected simple graph Γ , where a collection of nodes is V(Γ) , and the collection of lines is E(Γ) . Consider a subset D of V(Γ) . For any node x∉D , there exists a node y∈D such that x and y are connected by a line, then D⊆V(Γ) is considered a dominating set of Γ . Refer to examples in Haynes (2017); Ahangar et al. (2022); Abdlhusein and Al-Harere (2021); Raju and Nayaka (2023) for further information on domination in graphs. For a dominating set $D=\left\{x_{1},x_{2},\ldots ,x_{r}\right\}$ , if $D-x_{i}$ is not a dominating set then D is known as the minimal dominating set (M.D.S). The total number of M.D.S’s of Γ is represented as $T_{p}\left(\Gamma \right)$ . DTIs are new degree-based topological indices introduced by Ahmed A. M. Hanan et al. (2021). These are based on the domination degree.

Definition 2.1 (Ahmed et al., 2021a) For every node vinV(Γ) , the domination degree of v is equal to the number of M.D.S.’s of Γ that contain v , represented by $d_{d_{v}}$ . The minimum and maximum domination degree of Γ is $\delta_{d}\left(\Gamma \right)=\delta_{d}=min\left\{d_{d_{v}}:v\in V\left(\Gamma \right)\right\}\;\text{and}\;\Delta_{d}\left(\Gamma \right)=\Delta_{d}=max\left\{d_{d_{v}}:v\in V\left(\Gamma \right)\right\}$ , respectively.

The definitions of the first, second, and modified first Zagreb DTIs (Ahmed A. M. Hanan et al., 2021) are as given in Equations 1–3:

$$DM1\left(\Gamma \right)=\underset{v\in V\left(\Gamma \right)}{\sum }d_{d_{v}}^{2}$$ (1)

$$DM2\left(\Gamma \right)=\underset{uv\in E\left(\Gamma \right)}{\sum }d_{d_{u}}d_{d_{v}}$$ (2)

$$DM1^{*}\left(\Gamma \right)=\underset{uv\in E\left(\Gamma \right)}{\sum }\left[d_{d_{u}}+d_{d_{v}}\right]$$ (3)

The following are the definitions of the forgotten, hyper and modified forgotten DTIs (Ahmed H. et al., 2021) as given in Equations 4–6:

$$DF\left(\Gamma \right)=\underset{v\in V\left(\Gamma \right)}{\sum }d_{d_{v}}^{3}$$ (4)

$$DH\left(\Gamma \right)=\underset{uv\in E\left(\Gamma \right)}{\sum }{\left[d_{d_{u}}+d_{d_{v}}\right]}^{2}$$ (5)

$$DF^{*}\left(\Gamma \right)=\underset{uv\in E\left(\Gamma \right)}{\sum }\left[d_{d_{u}}^{2}+d_{d_{v}}^{2}\right]$$ (6)

To derive different TI’s in the scientific realm, algebraic polynomials, the significant Hosoya polynomials, and others (Ahmad et al., 2018; Diudea, 2006; Diudea et al., 2008; Knor and Tratnik, 2023; Chou and Witek, 2014; Balasubramanian, 2023; Ibrahim et al., 2022; Masmali et al., 2023; Chen, 2023; Aziz et al., 2023; Ali et al., 2022), are essential tools. These polynomials, particularly the distance-based ones such as the Wiener (1947) and hyper-Wiener index (Cash et al., 2002), provide information about the structures of molecules. According to studies (Afzal et al., 2021; Deutsch et al., 2014; Rai et al., 2020; Rasool et al., 2023), the M-polynomial, which was developed in 2015 (Afzal et al., 2020), is notable for its ability to clarify degree-based graph invariants.

Using polynomial methods, the authors introduced and produced the domination topological indices (Shashidhara R. et al., 2023). In this study, $\phi_{d}$ -polynomial is used to evaluate the DTIs of the molecules.

The $\phi_{d}$ -polynomial is stated as $\phi_{d}\left(\Gamma ,\alpha ,\beta \right)=\sum_{\delta_{d}\leq k\leq l\leq \Delta_{d}}d_{d}m_{\left(k,l\right)}\alpha^{k}\beta^{l}$ , where $d_{d}m_{\left(k,l\right)}\left(\Gamma \right)=|\left\{e=uv:d_{d_{u}}=k,d_{d_{v}}=l\right\}|$ .

The domination (D) indices on E(Γ) can be expressed as follows as give in Equation 7:

$$D\left(\Gamma \right)=\sum_{uv\in E\left(\Gamma \right)}f\left(d_{d_{u}},d_{d_{v}}\right)$$ (7)

Then, Table 1 gives the detailing of some DTIs. Hence, the DTIs from $\phi_{d}$ -polynomials are defined as in Table 2, in which $D_{\alpha }\left(f\left(\alpha ,\beta \right)\right)=\alpha \frac{\partial \left(f\left(\alpha ,\beta \right)\right)}{\partial \alpha }$ and $D_{\beta }\left(f\left(\alpha ,\beta \right)\right)=\beta \frac{\partial \left(f\left(\alpha ,\beta \right)\right)}{\partial \beta }$ .

**TABLE 1:** The detailing of some domination TI’s.

D indices	$f\left(d_{d_{u}},d_{d_{v}}\right)$
$DM1^{*}\left(\Gamma \right)$	$d_{d_{u}}+d_{d_{v}}$
$DF^{*}\left(\Gamma \right)$	$d_{d_{u}}^{2}+d_{d_{v}}^{2}$
DM2(Γ)	$d_{d_{u}}d_{d_{v}}$
DH(Γ)	${\left[d_{d_{u}}+d_{d_{v}}\right]}^{2}$

**TABLE 2:** Computation of domination TI’s from $\phi_{d}$ -polynomials.

D indices	Computation from $\phi_{d}\left(\Gamma \right)$
$DM1^{*}\left(\Gamma \right)$	$\left(D_{\alpha }+D_{\beta }\right)\left(\phi_{d}\left(\Gamma \right)\right){|}_{\alpha =\beta =1}$
$DF^{*}\left(\Gamma \right)$	$\left(D_{\alpha }^{2}+D_{\beta }^{2}\right)\left(\phi_{d}\left(\Gamma \right)\right){|}_{\alpha =\beta =1}$
DM2(Γ)	$\left(D_{\alpha }D_{\beta }\right)\left(\phi_{d}\left(\Gamma \right)\right){|}_{\alpha =\beta =1}$
DH(Γ)	${\left(D_{\alpha }+D_{\beta }\right)}^{2}\left(\phi_{d}\left(\Gamma \right)\right){|}_{\alpha =\beta =1}$

In this article, the DTIs of Γ of some heart disease drugs via $\phi_{d}$ -polynomials are derived.

## 3 Method and material used for computations

In this study, two primary computations are conducted: evaluating the DTIs and analyzing statistical parameters. Given that each descriptor is additive, results are obtained by summing the terms. The indices DM $1^{*}\left(\Gamma \right)$ , ${\text{DF}}^{*}\left(\Gamma \right)$ , DM 2(Γ) , DH (Γ) , DM 1(Γ) , and DF (Γ) are calculated using the node and line partition method based on node domination degrees. These partitions are then used to derive the $\phi_{d}$ -polynomials. Python software is employed for verification of these calculations, while scientific methods are applied. Experimental data for heart disease drugs are sourced from the chemical sites ChemSpider, PubChem and pkCSM. Regression models use the DTIs and experimental values as inputs. SPSS software manages the statistical computations, and Microsoft Excel can be used for regression calculations. The steps involved in using regression modeling and topological indices to analyze the properties of heart disease drugs are as follows:

• Domination Degree Calculation: Calculate the domination degrees for each node and partition the lines of each heart disease drug structure accordingly.

• DTI Calculation: Compute the DTIs by inserting the values of each line partition into the equations of the $\phi_{d}$ -polynomial.

• Data Collection: Obtain experimental values for the physicochemical properties of heart disease drugs from ChemSpider and PubChem, and ADMET properties from pkCSM.

• Regression Modeling: Develop inverse curvilinear and multilinear regression models using both estimated and experimental values.

• Statistical Analysis: Use SPSS software to analyze the relationship between experimental and estimated values, and determine correlations between the indices and properties.

• Descriptor Significance: Identify molecular descriptors with strong correlations as significant, while those with weak correlations are deemed less effective in describing the properties of heart disease drug structures.

## 4 Main results

**FIGURE 1:**
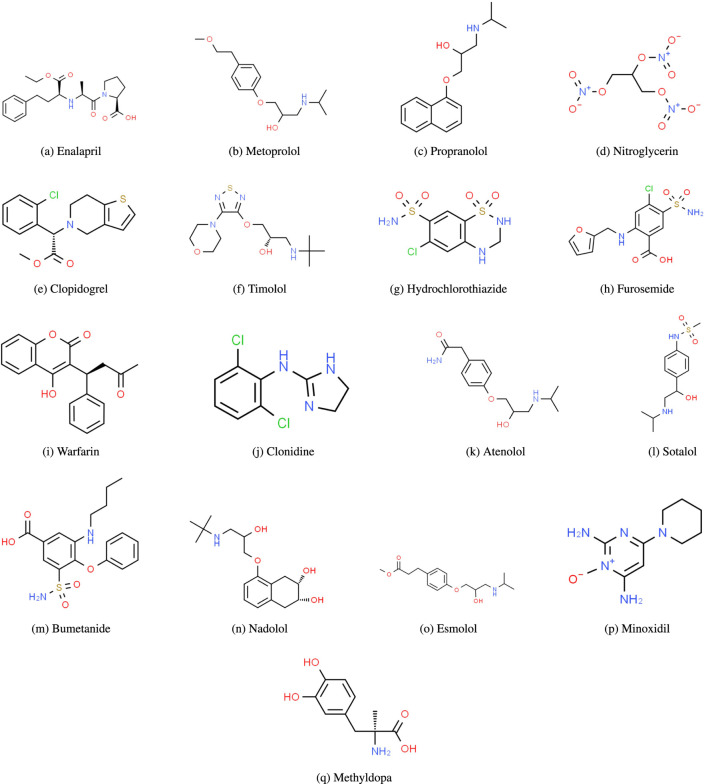
Molecular structures of heart disease drugs. **(A)** Enalapril. **(B)** Metoprolol, **(C)** Propranolol, **(D)** Nitroglycerin, **(E)** Clopidogrel, **(F)** Timolol, **(G)** Hydrochlorothiazide, **(H)** Furosemide, **(I)** Warfarin, **(J)** Clonidine, **(K)** Atenolol, **(L)** Sotalol, **(M)** Bumetanide, **(N)** Nadolol, **(O)** Esmolol, **(P)** Minoxidil, **(Q)** Methyldopa.

In this section, the above mentioned DTIs are evaluated for 17 heart disease drugs (see Figure 1) using $\phi_{d}$ -polynomials.

**FIGURE 2:**
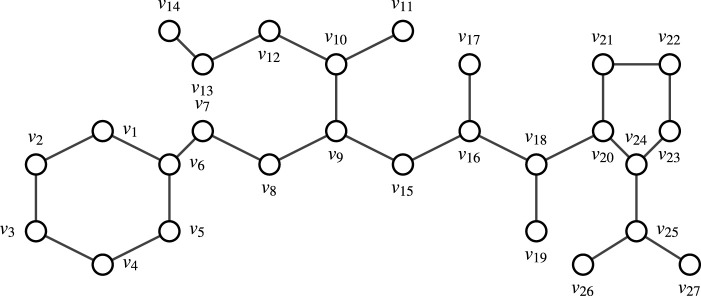
Molecular graph of Enalapril.

Consider Enalapril. Let Γ be the molecular graph of Enalapril. Figure 2 represent Γ with order 27 and size 28. To compute the total number of M.D.S’s for Γ , start by splitting Γ into two components $S_{1}$ and $S_{2}$ with nodes $\left\{v_{1},v_{2},v_{3},v_{4},v_{5},v_{6},v_{7},v_{8},v_{9},v_{10},v_{11},v_{12},v_{13},v_{14}\right\}$ and $\left\{v_{9},v_{15},v_{16},v_{17},v_{18},v_{19},v_{20},v_{21},v_{22},v_{23},v_{24},v_{25},v_{26},v_{27}\right\}$ , respectively. First, compute the number of M.D.S’s for each component, obtaining $T_{p}\left(S_{1}\right)=56$ and $T_{p}\left(S_{2}\right)=84$ . Then, combine every M.D.S of $S_{1}$ with every M.D.S of $S_{2}$ , resulting in 56×84=4704 potential sets. Next, verify the minimality of these sets by removing redundant nodes: if a node $v_{i}$ in a combined set is redundant (i.e., $v_{i}$ is in a minimal dominating set of $S_{1}$ and is adjacent to any node in $S_{2}$ ), then $v_{i}$ is removed. After checking and removing redundancies, the total number of M.D.S’s for Γ is $T_{p}\left(\Gamma \right)=3150$ .

Remark 4.1 Finding all M.D.S’s in a graph with more than 10 nodes manually is challenging. Therefore, one can use Algorithm 1 to identify all M.D.S’s and determine the node appearance count in the graph. This approach leverages a combination of brute-force methods and verification steps, specifically tailored for scenarios where the graph size is manageable. The corresponding Python code for this algorithm is available on GitHub.

By evaluating the domination degree of each node of Γ , the following Tables 3, 4 are obtained.

Let $d_{d}m_{\left(k,l\right)}\left(\Gamma \right)=|\left\{e=uv:d_{d_{u}}=k,d_{d_{v}}=l\right\}|$ . Based on the domination degree of each line’s end nodes, the line set of Γ is divided into 19 partitions, as shown in Table 4. The expression $\phi_{d}\left(\Gamma ,\alpha ,\beta \right)=\underset{\delta_{d}\leq k\leq l\leq \Delta_{d}}{\sum }d_{d}m_{\left(k,l\right)}\alpha^{k}\beta^{l}$ is then evaluated, serving as Supplementary Equation S1. The DTIs of Γ are evaluated from the $\phi_{d}$ -polynomial using the Python code, which is available at GitHub. The computed values are

$$DM1^{*}\left(\Gamma \right)=74403,\;DF^{*}\left(\Gamma \right)=101375127,\;DM2\left(\Gamma \right)=49431141,\;DH\left(\Gamma \right)=200237409.$$

Also, $DM1\left(\Gamma \right)=\underset{v\in V\left(\Gamma \right)}{\sum }d_{d_{v}}^{2}=51874146$ and $DF\left(\Gamma \right)=\underset{v\in V\left(\Gamma \right)}{\sum }d_{d_{v}}^{3}=74699221950$ .

**TABLE 3:** $d_{d_{v}}$ of each node of Γ .

$d_{d_{v}}$	840	1050	1134	1176	1260	1350	1386	1428	1470
Number of nodes	1	4	1	1	2	3	4	1	1
$d_{d_{v}}$	1500	1575	1650	1680	1764	-	-	-	-
Number of nodes	2	2	3	1	1	-	-	-	-

**TABLE 4:** Line division according to the domination degree of the end nodes of each line.

(k,l)	(840, 1470)	(840, 1575)	(1050, 1386)	(1050, 1428)	(1050,1050)
$d_{d}m_{\left(k,l\right)}$	1	1	3	1	1
(k,l)	(1050,1350)	(1050, 1500)	(1134, 1260)	(1134, 1428)	(1176, 1260)
$d_{d}m_{\left(k,l\right)}$	2	2	1	1	2
(k,l)	(1176, 1386)	(1350, 1350)	(1386, 11764)	(1386, 1500)	(1260, 1386)
$d_{d}m_{\left(k,l\right)}$	1	2	1	1	2
(k,l)	(1428, 1470)	(1575, 1575)	(1500, 1650)	(1470, 1680)	-
$d_{d}m_{\left(k,l\right)}$	1	1	3	1	-

Algorithm 1. Finding Minimal Dominating Sets and Node Appearance Count.

**Require:** A graph Γ=(V,E)

**Ensure:** A list of minimal dominating sets in Γ and a count of node appearances
  **Check if a set is a dominating set**
   Initialize nodes_covered as the set of all nodes in S and their neighbors.
  Return **True** if nodes_covered=V , otherwise **False**.
  **Check if a set is a minimal dominating set**
   **if** S is not a dominating set **then**
     Return **False**.
   **end if**
   **for** each node u in S **do**
     Create a copy of S , called $S^{\prime }$ .
     Remove u from $S^{\prime }$ .
     **if** $S^{\prime }$ is a dominating set **then**
       Return **False**.
     **end if**
   **end for**
   Return **True**.
   **Find all minimal dominating sets**
   Initialize minimal_dominating_sets as an empty list.
   **for** each r from 1 to |V| **do**
     Generate all combinations of r nodes from V .
     **for** each combination S **do**
       **if** S is a minimal dominating set **then**
       Add S to minimal_dominating_sets .
      **end if**
    **end for**
  **end for**
  Return minimal_dominating_sets .
   **Count vertex appearances in minimal dominating sets**
   Initialize node_count with each node in V having a count of 0.
   **for** each minimal dominating set S in minimal_dominating_sets **do**
     **for** each node u in S **do**
       Increment node_count[u] by 1.
     **end for**
   **end for**
   Return node_count .
   **Execution**
 Call the procedure to find all minimal dominating sets.
   Call the procedure to count node appearances.
   Return the list of minimal dominating sets and the node appearance counts.

**FIGURE 3:**
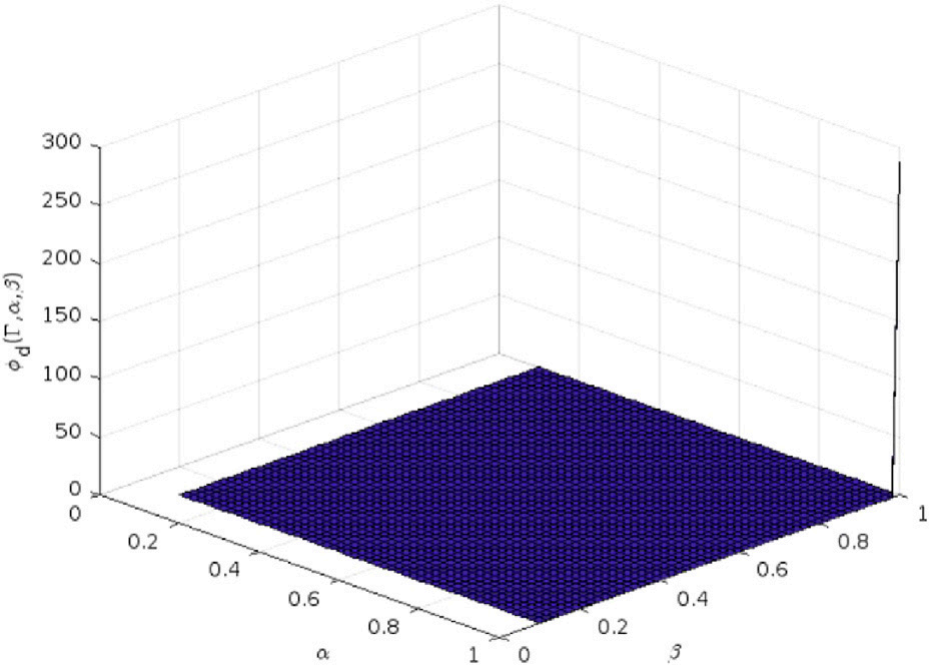
Plotting of $\phi_{d}$ -polynomial of enalapril.

The 3D plot of the $\phi_{d}$ -polynomial for Enalapril, depicted in Figure 3, is generated using MATLAB. The analysis reveals that the values obtained from the $\phi_{d}$ -polynomial exhibit distinct behaviors in relation to the parameters α and β . By adjusting these parameters, one can effectively control the values of the $\phi_{d}$ -polynomial. Using the method described for the drug Enalapril, the DTIs are computed for 16 additional heart disease drugs. Table 5 presents the computed data for these indices.

**TABLE 5:** DTIs of 17 heart disease drugs.

Drugs	DM1^*^	DF^*^	DM2	DH	DM1	DF
Enalapril	74403	101375127	49431141	200237409	51874146	74699221950
Metoprolol	5174	732078	347169	1426416	346940	51071346
Propranolol	5042	646492	318215	1282922	333947	45987239
Nitroglycerin	597	12945	25753	6404	7509	171591
Clopidogrel	18142	7272140	3585416	14442972	3541100	1496155096
Timolol	7614	1345370	659611	2664592	718478	134875043
Hydrochlorothiazide	1332	51844	24028	99900	23696	932074
Furosemide	7365	1270449	613062	2496573	611755	107769039
Warfarin	13878	4078100	1882880	7843860	1925380	593539224
Clonidine	1319	58769	28837	116443	27403	1238517
Atenolol	3541	341349	163097	667543	190242	17337492
Sotalol	1572	71738	33910	139558	41067	2075401
Bumetanide	28551	16118757	7795211	31709179	8121561	4569726806
Nadolol	11246	2800530	1378695	5557920	1488290	401836542
Esmolol	11792	3411104	1652216	6715536	1812712	552344512
Minoxidil	1200	46840	22476	91792	21656	862976
Methyldopa	795	21497	10592	42681	10527	294615

## 5 Curvilinear regression analysis of heart disease drugs

Numerous statistical methods are used in regression analysis to calculate the correlations between a dependent variable and multiple independent variables. There are several varieties of this technique, including multiple linear, nonlinear, and linear regression. Among the most popular models are multiple linear regression and simple linear regression. However, nonlinear regression analysis is better suitable when there is a nonlinear relationship between the variables. Simple linear, quadratic, and cubic regression are all performed using the following model Equations 8–10 (Roustaei, 2024).

$$P=a_{1}\left(DTI\right)+b$$ (8)

$$P=a_{1}{\left(DTI\right)}^{2}+a_{2}\left(DTI\right)+b$$ (9)

$$P=a_{1}{\left(DTI\right)}^{3}+a_{2}{\left(DTI\right)}^{2}+a_{3}\left(DTI\right)+b$$ (10)

Here, DTI represents the independent variable, P (which could be a physicochemical or ADMET property) is the dependent variable, $a_{i};1\leq i\leq 3$ are the regression coefficients, and b is the constant term. To obtain the best fitting model, we considered the inverse of the DTI values and the physicochemical or ADMET properties in this study. The following model Equations 11–13 are used in this analysis.

$$\frac{1}{P}=a_{1}\left(\frac{1}{DTI}\right)+b$$ (11)

$$\frac{1}{P}=a_{1}{\left(\frac{1}{DTI}\right)}^{2}+a_{2}\left(\frac{1}{DTI}\right)+b$$ (12)

$$\frac{1}{P}=a_{1}{\left(\frac{1}{DTI}\right)}^{3}+a_{2}{\left(\frac{1}{DTI}\right)}^{2}+a_{3}\left(\frac{1}{DTI}\right)+b$$ (13)

Multivariate regression is the most common type of linear regression. It shows how a single dependent variable is linearly correlated with multiple independent variables. The model is as follows in Equation 14 (Roustaei, 2024):

$$\frac{1}{P}=\overset{n}{\underset{i=1}{\sum }}a_{i}{\left(\frac{1}{DTI}\right)}^{i}+b.$$ (14)

Where, $a_{i};1\leq i\leq n$ are the regression coefficients and b is the constant term.

Table 5 presents the calculated DTI values for the selected heart disease drugs, which were determined using Python software. The experimental data for the drugs are obtained from ChemSpider and PubChem and are detailed in Table 6. This data includes Molar Refraction (MR), Polarizability (P), Molar Volume (MV), Melting Point (MP), and Molecular Weight (MW). Additionally, the experimental values for the ADMET properties of the drugs are sourced from pkCSM and are listed in the Table 7. These values encompass Oral Rat Chronic Toxicity (OC), Caco2 Permeability (Caco2), Skin Permeability (SKIN), Intestinal absorption (IA), and T. Pyriformis toxicity (TP).

**TABLE 6:** Physicochemical properties of drugs for heart disease.

Drugs	MR	P	MV	MP	MW
Enalapril	99.5	39.5	312.6	143	376.4
Metoprolol	77.1	30.6	258.7	120	267.36
Propranolol	79	31.3	237.2	96	259.34
Nitroglycerin	39.2	15.5	135.9	13.5	227.09
Clopidogrel	85.5	33.9	244.3	158	321.8
Timolol	82.2	32.6	258.5	71.5	316.42
Hydrochlorothiazide	62.7	24.9	175.8	273	297.7
Furosemide	75.8	30	205.8	206	330.74
Warfarin	84.4	33.5	235.8	161	308.3
Clonidine	57.3	22.7	153.2	130	230.09
Atenolol	74.3	29.4	236.7	146	266.34
Sotalol	72.1	28.6	219.7	206.5	272.37
Bumetanide	94.1	37.3	270.5	230	364.4
Nadolol	85.8	34	260	124	309.4
Esmolol	81.7	32.4	272.4	-	295.37
Minoxidil	54.6	21.6	137.6	-	209.25
Methyldopa	53.9	21.4	150.5	300	211.21

**TABLE 7:** ADMET properties of drugs for heart disease.

Drugs	OC	Caco2	SKIN	IA	TP
Enalapril	2.099	0.334	−2.735	43.99	0.285
Metoprolol	1.606	1.387	−2.831	90.657	1.151
Propranolol	1.762	1.629	−2.783	92.304	1.073
Nitroglycerin	0.049	−0.279	−2.531	68.208	0.216
Clopidogrel	1.436	1.608	−2.597	90.84	0.352
Timolol	1.64	1.054	−2.955	86.261	0.257
Hydrochlorothiazide	2.105	0.35	−2.901	72.659	0.336
Furosemide	1.97	−0.157	−2.735	61.358	0.285
Warfarin	1.081	0.928	−2.754	96.161	0.591
Clonidine	1.438	1.48	−2.729	89.872	0.406
Atenolol	2.22	0.632	−3.022	67.901	0.509
Sotalol	1.655	0.985	−2.938	77.294	0.484
Bumetanide	1.737	0.939	−2.735	65.845	0.285
Nadolol	2.366	0.521	−2.89	65.391	0.246
Esmolol	2.163	0.661	−2.908	90.35	1.078
Minoxidil	1.098	0.653	−2.798	94.641	0.017
Methyldopa	1.995	−0.039	−2.735	44.13	0.285

Table 8 depicts the correlation coefficient (R) between these TIs and the physicochemical properties of the drugs, derived from inverse linear regression analyses. The highest R value is emphasized in bold. Table 9 outlines the optimal linear regression equations for approximating the physicochemical properties of these drugs, characterized by the maximum R value, the minimum standard error (SE), the maximum F value and significance (P) value less than .10. Table 10 displays the R obtained from quadratic regression analyses, with the highest R value emphasized in bold. Table 11 presents the quadratic regression equations that best approximate the physicochemical properties of the drugs studied. Furthermore, Table 12 shows the R from cubic regression analyses, and Table 13 lists the corresponding cubic regression equations.

**TABLE 8:** The correlation coefficient (R) determined using linear regression models.

Domination index	1/MR	1/P	1/MV	1/MP	1/MW
1/DM1^*^	**0.964**	**0.963**	**0.905**	0.638	**0.796**
1/DF^*^	0.948	0.947	0.822	0.766	0.685
1/DM2	0.724	0.722	0.812	0.151	0.767
1/DH	0.838	0.839	0.602	**0.963**	0.435
1/DM1	0.95	0.949	0.857	0.703	0.722
1/DF	0.92	0.92	0.771	0.795	0.633

**TABLE 9:** Linear regression equations offer the most precise estimates of physicochemical properties.

Linear regression equation	R	F	SE	P
$\frac{1}{MR}$ = 7.658 $\left(\frac{1}{DM1^{*}}\right)$ + 0.011	0.964	198.233	0.001	< 0.001
$\frac{1}{P}$ = 19.367 $\left(\frac{1}{DM1^{*}}\right)$ + 0.028	0.963	192.634	0.003	< 0.001
$\frac{1}{MV}$ = 2.504 $\left(\frac{1}{DM1^{*}}\right)$ + 0.004	0.905	67.478	0.001	< 0.001
$\frac{1}{MP}$ = 424.657 $\left(\frac{1}{DF}\right)$ + 0.005	0.963	166.296	0.005	< 0.001
$\frac{1}{MW}$ = 1.062 $\left(\frac{1}{DM1^{*}}\right)$ + 0.003	0.796	25.897	0.000	< 0.001

**TABLE 10:** The correlation coefficient (R) determined using quadratic regression models.

Domination index	1/MR	1/P	1/MV	1/MP	1/MW
1/DM $1^{*}$	**0.968**	**0.967**	0.921	0.893	**0.845**
1/DF^*^	0.953	0.952	0.909	0.962	0.807
1/DM2	0.826	0.824	0.898	0.423	0.796
1/DH	0.953	0.953	0.884	**0.990**	0.793
1/DM1	0.951	0.950	**0.924**	0.919	0.806
1/DF	0.932	0.931	0.885	0.974	0.780

**TABLE 11:** Quadratic regression equations offer the most precise estimates of physicochemical properties.

Quadratic regression equation	R	F	SE	P
$\frac{1}{MR}$ = 1443.558 ${\left(\frac{1}{DM1^{*}}\right)}^{2}$ + 5.553 $\left(\frac{1}{DM1^{*}}\right)$ + 0.011	0.968	104.346	0.001	< 0.001
$\frac{1}{P}$ = 3725.182 $\left({\frac{1}{DM1^{*}}}^{2}\right)$ + 13.9333 $\left(\frac{1}{DM1^{*}}\right)$ + 0.028	0.967	101.577	0.003	< 0.001
$\frac{1}{MV}$ = −322703.072 ${\left(\frac{1}{DM1}\right)}^{2}$ + 67.148 $\left(\frac{1}{DM1}\right)$ + 0.004	0.924	40.924	0.001	< 0.001
$\frac{1}{MP}$ = 4622930.313 ${\left(\frac{1}{DH}\right)}^{2}$ - 296.996 $\left(\frac{1}{DH}\right)$ + 0.008	0.990	311.123	0.003	< 0.001
$\frac{1}{MW}$ = −793.034 ${\left(\frac{1}{DM1^{*}}\right)}^{2}$ + 2.219 $\left(\frac{1}{DM1^{*}}\right)$ + 0.003	0.845	17.407	0.000	< 0.001

**TABLE 12:** The correlation coefficient (R) determined using cubic regression models.

Domination index	1/MR	1/P	1/MV	1/MP	1/MW
1/DM1^*^	**0.977**	**0.977**	0.923	0.982	**0.845**
1/DF^*^	0.97	0.97	0.921	**0.991**	0.807
1/DM2	0.827	0.825	0.91	0.471	0.808
1/DH	0.953	0.953	0.884	0.99	0.793
1/DM1	0.973	0.973	0.93	0.99	0.806
1/DF	0.97	0.97	**0.935**	**0.991**	0.788

The numbers in bold are the highest R values in each property.

**TABLE 13:** Cubic regression equations offer the most precise estimates of physicochemical properties.

Cubic regression equation	R	F	SE	P
$\frac{1}{MR}$ = 6534284.602 ${\left(\frac{1}{DM1^{*}}\right)}^{3}$ - 14629.820 ${\left(\frac{1}{DM1^{*}}\right)}^{2}$ + 15.024 $\left(\frac{1}{DM1^{*}}\right)$ + 0.011	0.977	92.912	0.001	< 0.001
$\frac{1}{P}$ = 16853876.058 ${\left(\frac{1}{DM1^{*}}\right)}^{3}$ - 37732.872 ${\left(\frac{1}{DM1^{*}}\right)}^{2}$ + 38.361 $\left(\frac{1}{DM1^{*}}\right)$ + 0.027	0.977	90.979	0.002	< 0.001
$\frac{1}{MV}$ = 1.185E+14 ${\left(\frac{1}{DF}\right)}^{3}$ - 1186151596 ${\left(\frac{1}{DF}\right)}^{2}$ + 3479.016 $\left(\frac{1}{DF}\right)$ + 0.004	0.935	30.376	0.001	< 0.001
$\frac{1}{MP}$ = 9.402E+14 ${\left(\frac{1}{DF}\right)}^{3}$ - 3469413686 ${\left(\frac{1}{DF}\right)}^{2}$ - 305.448 $\left(\frac{1}{DF}\right)$ + 0.008	0.991	192.588	0.003	< 0.001
$\frac{1}{MP}$ = 5.019E+11 ${\left(\frac{1}{DF^{*}}\right)}^{3}$ - 31128294.957 ${\left(\frac{1}{DF^{*}}\right)}^{2}$ + 272.756 $\left(\frac{1}{DF^{*}}\right)$ + 0.007	0.991	198.214	0.003	< 0.001
$\frac{1}{MW}$ = 265571.449 ${\left(\frac{1}{DM1^{*}}\right)}^{3}$ - 1446.300 ${\left(\frac{1}{DM1^{*}}\right)}^{2}$ + 2.604 $\left(\frac{1}{DM1^{*}}\right)$ + 0.003	0.845	10.833	0.000	< 0.001

The table compares R from inverse linear, quadratic, and cubic regression models, analyzing relationships between domination indices ( ${\text{DM}1}^{*}$ , ${\text{DF}}^{*}$ , DM2, DH, DM1, DF) and physicochemical properties (MR, P, MV, MP, MW). It highlights the trends in correlations under different regression models.

For the linear regression model, ${\text{DM}1}^{*}$ exhibits the strongest correlations with MR (R=0.964) and P (R=0.963) , indicating strong linear relationships. Other properties, such as MV (R = 0.905) and MP (R = 0.638), also exhibit good correlations. DH achieves a notable correlation with MP (R=0.963) , but indices like DM2 perform poorly, especially with MP (R=0.151) . These results suggest that inverse linear models are effective for predicting the physicochemical properties of heart disease drugs.

The quadratic regression model improves correlations, capturing nonlinear relationships. For ${\text{DM}1}^{*}$ , R increases to 0.968 and 0.967 for MR and P, respectively. DH shows an excellent correlation with MP (R=0.990) , while DM1 performs well for MV (R=0.924) . However,DM2 exhibits weaker performance with MP (R = 0.423). Overall, inverse quadratic models prove effective for predicting the physicochemical properties of heart disease drugs.

The cubic regression model provides the best overall fit, achieving the highest correlations across most combinations. For ${\text{DM}1}^{*}$ , the cubic model achieves R=0.977 for both MR and P. ${\text{DF}}^{*}$ and DM1 exhibit near-perfect correlations with MP (R=0.991) . DM1 also shows strong correlations with MV (R=0.930) . Even DM2, which performed poorly in earlier models, shows slight improvement under the cubic model. This highlights the cubic model’s ability to handle complex relationships. Key insights derived from Tables 8–13:

• Among the six domination indices, ${\text{DM}1}^{*}$ and DF are the most effective for predicting properties like MR, P, MV, MP, and MW.

• ${\text{DM}1}^{*}$ provides the best MR models with R=0.964 , 0.968, and 0.977 for linear, quadratic, and cubic regression models, respectively. A close alternative MR model is derived from ${\text{DF}}^{*}$ , DM1, and DF with R=0.97 in the cubic model.

• ${\text{DM}1}^{*}$ is the best predictor for P, achieving R=0.963 , 0.967, and 0.977 across the three models. A close alternative cubic model is derived from ${\text{DF}}^{*}$ , DH, DM1, and DF, with R=0.97 .

• The best cubic model for MV comes from DF (R=0.935) , followed closely by DM1 (R=0.93) . Linear and quadratic models show the best fit with ${\text{DM}1}^{*}$ (R=0.905) and DM1 (R=0.924) .

• The cubic regression models of ${\text{DF}}^{*}$ and DF provide the best correlations (R=0.991) . For linear and quadratic models, DH shows strong performance ( R=0.963 and R=0.990 ).

• ${\text{DM}1}^{*}$ offers the best MW models, with R=0.796 , 0.845, and 0.845 across the three models.

The cubic regression model demonstrates consistent superiority over linear and quadratic models in predicting the physicochemical properties of heart disease drugs. However, both linear and quadratic models also show strong predictive performance and closely approximate the observed physicochemical properties in several cases. Among the indices, ${\text{DM}1}^{*}$ and DM1 emerge as the most reliable predictors, especially for properties like MR, P, and MP.

The comparison of actual and predicted values for physicochemical properties using inverse linear, quadratic, and cubic regression models (Supplementary Tables S1–S3) highlights the predictive accuracy of the proposed models. All regression models demonstrate a strong correlation between actual and predicted values for most physicochemical properties, such as MR, P, MP, and MW. However, their accuracy varies depending on the specific property and regression type. For instance, in the case of MR for drugs like Enalapril and Metoprolol, the predictions from linear, quadratic, and cubic regression models exhibit close alignment with the actual values.

The results from Table 14 highlight the efficacy of cubic regression models in correlating DTIs with various ADMET properties of heart disease drugs. Among the indices, DF and DH consistently exhibit high predictive accuracy, with nearly perfect correlations (R > 0.99) for properties like OC, demonstrating their reliability in such analyses. While DF stands out as the best predictor for Caco2 permeability (R = 0.951), indices like DM2, ${\text{DF}}^{*}$ , and DM1 also show strong correlations, making them suitable alternatives. For skin permeability, DM $1^{*}$ achieves the highest correlation (R = 0.718), indicating its dominance for this property, whereas ${\text{DF}}^{*}$ is the best fit for IA with R = 0.622. Interestingly, DM2 outperforms other indices for TP with R = 0.673, emphasizing its unique predictive capacity for this property.

**TABLE 14:** The correlation coefficient (R) between the computed DTIs and ADMET properties determined using cubic regression models.

Domination index	1/OC	1/Caco2	1/SKIN	1/IA	1/TP
1/DM1^*^	0.982	0.714	**0.718**	0.619	0.422
1/DF^*^	0.999	0.938	0.625	**0.622**	0.454
1/DM2	0.41	0.942	0.375	0.606	**0.673**
1/DH	**1**	0.718	0.588	0.354	0.299
1/DM1	0.996	0.914	0.618	0.612	0.463
1/DF	0.999	**0.951**	0.604	0.621	0.532

The numbers in bold are the highest R values in each property.

These findings underscore the ability of DTIs to serve as robust structural predictors, particularly for OC and Caco2, while suggesting the need for further exploration of weaker correlations, such as those observed with DM2 for OC and DH for TP. The strong performance of DF in predicting both OC and Caco2 permeability supports the hypothesis that specific domination indices may reflect critical molecular features influencing drug absorption and permeability. Additionally, the moderate correlations for properties like skin permeability and IA indicate that while cubic regression captures significant relationships.

In conclusion, the cubic regression model effectively captures the complex relationships between DTIs and ADMET properties, offering a robust framework for predicting critical drug characteristics. Table 15 highlights the most effective cubic regression models for predicting these properties. The strong correlations observed between most ADMET properties and DTIs underscore the reliability of these indices as predictors.

**TABLE 15:** Cubic regression equations offer the most precise estimates of ADMET properties.

Regression equation	R	F	SE	P
$\frac{1}{OC}$ = 1.439E+14 ${\left(\frac{1}{DF^{*}}\right)}^{3}$ - 9514352004 ${\left(\frac{1}{DF^{*}}\right)}^{2}$ + 133711.664 $\left(\frac{1}{DF^{*}}\right)$ + 0.486	0.999	2214.038	0.236	< 0.001
$\frac{1}{OC}$ = 3.006E+17 ${\left(\frac{1}{DF}\right)}^{3}$ - 1.370E+12 ${\left(\frac{1}{DF}\right)}^{2}$ + 1183585.413 $\left(\frac{1}{DF}\right)$ + 0.551	0.999	3667.509	0.183	< 0.001
$\frac{1}{Caco2}$ = 1.345E+18 ${\left(\frac{1}{DF}\right)}^{3}$ - 9.566E+12 ${\left(\frac{1}{DF}\right)}^{2}$ + 9382583.380 $\left(\frac{1}{DF}\right)$ + 0.406	0.951	41.158	2.311	< 0.001
$\frac{1}{SKIN}$ = 47795612.252 ${\left(\frac{1}{DM1^{*}}\right)}^{3}$ - 159034.158 ${\left(\frac{1}{DM1^{*}}\right)}^{2}$ + 117.532 $\left(\frac{1}{DM1^{*}}\right)$ - 0.368	0.718	4.622	0.013	0.021
$\frac{1}{IA}$ = −2.719E+11 ${\left(\frac{1}{DF^{*}}\right)}^{3}$ + 28042144.698 ${\left(\frac{1}{DF^{*}}\right)}^{2}$ - 535.8550 $\left(\frac{1}{DF^{*}}\right)$ + 0.014	0.622	2.727	0.003	0.087
$\frac{1}{TP}$ = −9.709E+14 ${\left(\frac{1}{DM2}\right)}^{3}$ + 1.242E+11 ${\left(\frac{1}{DM2}\right)}^{2}$ - 3095176.302 $\left(\frac{1}{DM2}\right)$ + 6.433	0.673	3.580	11.213	0.044

To further enhance prediction accuracy, future studies could explore the role of specific molecular features represented by DTIs and investigate alternative modeling techniques. Overall, the results demonstrate that DTIs are valuable tools for modeling the ADMET properties of heart disease drugs.

## 6 Multivariate regression analysis

Supplementary Equations S2–S6 represent the best-fitting multivariate regression models for the five physicochemical properties of heart disease drugs. Table 16 presents the statistical parameters between the computed DTIs and the physicochemical properties of heart disease drugs using multilinear regression models. The key statistical values displayed in this table are:

• This shows a very high positive correlation for the properties listed, such as MR, P, MV, MP, and MW, all with values greater than 0.93. These high R values suggest that the proposed multilinear regression models are highly effective in predicting the corresponding properties.

• The R^2^ values indicate how well the regression models explain the variance in the physicochemical properties. For most properties, R^2^ values are very close to 1, particularly for MR (0.9889) and P (0.9886), signifying that the models can explain over .98.

• The SE values are quite low, especially for MR (5.15E-04), P (0.00132), and MV (4.19E-04), indicating a high level of accuracy in the predictions made by the models.

• The F values are consistently high, particularly for MR (148.7425) and P (144.8956), implying that the models significantly explain the variability in the data compared to random chance.

• All p-values are less than 0.0001, which indicates that the relationships between the computed DTIs and the physicochemical properties are statistically significant.

**TABLE 16:** The statistical parameters between the computed DTIs and physicochemical properties of heart disease drugs using multilinear regression models.

Property	R	R^2^	SE	F	P
MR	0.994	0.9889	5.15E-04	148.7425	< 0.0001
P	0.994	0.9886	0.00132	144.8956	< 0.0001
MV	0.969	0.9396	4.19E-04	25.91746	< 0.0001
MP	0.993	0.9861	0.00274	94.3163	< 0.0001
MW	0.939	0.8821	2.82E-04	12.47079	3.89E-04

Supplementary Table S4 provides a comparison of the actual and predicted values for the physicochemical properties (MR, P, MV, MP, MW) of the heart disease drugs using the multilinear regression models. This Table reveals the following observations.

• The predicted values are very close to the actual values for most drugs. For example, for Enalapril, the predicted MR value (0.01064) is very close to the actual value (0.01005), and similarly, for Metoprolol, the predicted P value (0.03312) closely matches the actual value (0.03268). These minor discrepancies suggest that the multilinear regression models have strong predictive power.

• For some drugs, such as Nitroglycerin and Warfarin, the predictions are very accurate across all properties, with values almost identical to the actual ones. This indicates that the model is particularly robust for certain drugs. However, for Minoxidil and Esmolol, the prediction for MP and MV has some variability, but the predictions are still relatively close to the actual values.

• In general, the model consistently predicts the physicochemical properties with minimal deviation from the actual values. The use of multilinear regression models appears to offer a reliable approach to predict the properties of heart disease drugs.

The results in Table 16 confirm that the multilinear regression models are highly effective in capturing the physicochemical properties of heart disease drugs, as evidenced by the very high R and R^2^ values, low SE, high F-statistics, and statistically significant p-values. Furthermore, the analysis of Supplementary Table S4 demonstrates that the model’s predictions for the various physicochemical properties are remarkably accurate, affirming the model’s strong predictive power. This highlights the effectiveness of the proposed regression models in drug property prediction and their potential for further applications in computational drug design.

The best-fitting multivariate regression models for the ADMET properties of heart disease drugs are presented in Supplementary Equations S7–S11. The multilinear regression models for predicting the ADMET properties of heart disease drugs, presented in Table 17, exhibit strong performance for certain properties while being less effective for others. The model for OC demonstrates exceptional accuracy, with an R value of 0.999, a low standard error, and a highly significant F-statistic, indicating near-perfect prediction. Similarly, the model for Caco-2 permeability performs well, achieving an R value of 0.961. In contrast, the models for SKIN, IA, and TP show varying levels of effectiveness. The SKIN model provides a good fit with an R value of 0.812, while the IA and TP models, with R values of 0.704 and 0.603, respectively, exhibit moderate predictive power, particularly for TP. Overall, these results highlight the potential of multilinear regression models to predict ADMET properties of heart disease drugs, with varying degrees of accuracy depending on the specific property being analyzed.

**TABLE 17:** The statistical parameters between the computed DTIs and ADMET properties of heart disease drugs using multilinear regression models.

Property	R	SE	F	P
OC	0.999	0.161	2392.349	< 0.0001
Caco2	0.961	2.356	20.222	< 0.0001
SKIN	0.812	0.012	3.28647	0.04917
IA	0.704	0.003	1.644	0.2323
TP	0.603	13.781	0.9527	0.501

Although several studies have concentrated on the QSPR analysis of the physicochemical properties of heart disease drugs, there is no research on their ADMET properties. Additionally, our proposed cubic and multilinear regression models demonstrate a strong correlation between the indices and the ADMET properties of these drugs. Therefore, our model is valuable for predicting the ADMET properties of these drugs.

## 7 Discussion

This study offers significant improvements over traditional methods by enhancing both efficiency and accuracy. By using chemical graph theory and computational algorithms, it streamlines the evaluation of minimal dominating sets and computation of domination degree-based indices, reducing manual effort and potential errors. Unlike traditional methods that rely on time-consuming experimental measurements, the $\phi_{d}$ -polynomial and DTIs provide a computationally efficient framework that captures molecular structural properties. The integration of curvilinear and multilinear regression models further improves predictive accuracy, addressing nonlinear relationships often overlooked by traditional approaches. This method demonstrates superior scalability, reproducibility, and precision, making it a valuable tool for drug property prediction and molecular analysis, with potential for further validation on larger datasets and diverse drug classes.

Our study offers a significant advancement over existing QSPR investigations. In Hasani and Ghods (2023), QSPR analysis was conducted on four calcium channel-blocking heart treatment drugs, resulting in higher correlation values. However, it is not directly feasible to compare these regression models with our proposed models, which are based on a data set of 17 drug molecules. Similarly, the study (Hakeem et al., 2024) reports higher correlation coefficients for certain physicochemical properties compared to our models. Nevertheless, our models yield more accurate property predictions than the models referenced in these studies. This enhanced accuracy can be attributed to the extended dataset used in our analysis, which represents a significant increase compared to the existing datasets employed in previous QSPR models.

The carefully chosen 17 drug molecules not only provide a strong correlation between DTIs and drug properties but also reflect a diverse set of molecular structures, which enhances the generalizability of the resulting QSPR models. By incorporating drugs from different cardiovascular drug classes, such as antihypertensives, antiarrhythmic agents, and lipid-lowering medications, the selection allows for a deeper understanding of how variations in molecular architecture can influence key physicochemical and ADMET properties. This diversity ensures that the developed models are versatile and applicable to a wider range of compounds. Additionally, the focus on these 17 molecules enables efficient computation, which is critical for maintaining reproducibility and reliability in the predictive analysis. The use of DTIs as a core feature of the model further enhances its scientific value, offering valuable insights into the relationship between molecular structure and drug behavior. This approach also establishes a methodological framework that can be extended to future studies, promoting a more comprehensive understanding of drug properties across different classes and chemical spaces.

The inverse regression method is selected for its capability to effectively capture complex, nonlinear relationships between variables, providing greater flexibility compared to regression models. This method is particularly suited for scenarios where the relationship between independent and dependent variables is nonlinear, enabling more precise modeling. Using SPSS software, we implemented the inverse regression models (linear, quadratic and cubic) to calculate the constants and coefficients that minimize the error between observed and predicted values, ensuring a superior fit for the dataset. In QSPR analysis, the statistical evaluation incorporates R, SE, and P-value. The R value quantifies the strength of the relationship between variables, indicating the proportion of variance in the dependent variable explained by the model. SE measures the square root of the average squared differences between observed and predicted values, while a P-value less than 0.10 signifies the statistical significance of the model. An ideal regression model is characterized by a high R value, low SE, and a significant P-value. SPSS software is employed to calculate these metrics, ensuring accurate and reliable analysis.

The correlation coefficient (R) is chosen for this study because it effectively measures how well the regression model aligns with the data, providing a clear indication of the strength of relationships between variables. Its simplicity and widespread use in statistical analysis make it an ideal choice. In this work, the evaluation of DTIs is based on their correlation with selected physicochemical and ADMET properties, ensuring that indices with stronger correlations are prioritized. Previous studies, such as those by Gutman and Tošović (2013) and Sardar and Hakami (2024), have employed similar approaches to assess the ability of graphical indices to explain physicochemical characteristics. Our findings reveal that ${\text{DM}1}^{*}$ shows strong correlations with MW, P, MR, and SKIN; DF demonstrates excellent correlations with MV, MP, OC, and Caco2; ${\text{DF}}^{*}$ exhibits high correlations with MP and IA; and DM2 correlates strongly with TP. These results highlight the reliability of these indices as predictors of physicochemical and ADMET properties, underscoring their importance in QSPR modeling.

The practical interpretability of DTIs offers valuable insights for pharmaceutical applications, particularly in the design and optimization of drug candidates. DTIs, which quantify the structural properties of molecules by modeling them as graphs, provide a quantitative measure of how the connectivity and arrangement of atoms in a molecule can influence its physicochemical and pharmacokinetic properties. Medicinal chemists can use DTIs to gain a deeper understanding of the molecular structure of potential drug candidates and how these structures might influence important properties like solubility, stability, and permeability. By analyzing the relationship between specific topological features (such as connectivity, branching, or ring structure) and desired properties (e.g., boiling point, logP, or water solubility), chemists can prioritize molecular designs that are more likely to exhibit optimal characteristics. This enables more efficient identification of candidates with favorable physicochemical profiles, reducing the time and cost involved in the drug development process.

Extending DTIs to other therapeutic areas, such as antimicrobial, anticancer, or neurological drugs, could enhance their utility in drug discovery by predicting specialized properties required for different diseases. Incorporating domain-specific knowledge could improve predictions for drug properties like cellular uptake or receptor binding. Integrating DTIs with machine learning models, such as random forests or neural networks, offers the potential to capture complex, nonlinear relationships, improving prediction accuracy for drug properties, including ADMET profiles and efficacy. This integration could also enhance scalability, accelerating the drug discovery process and leading to more personalized therapeutic approaches.

### 7.1 Implications

DTIs have the potential to aid in predicting potential drug-drug interactions, and therefore improve the safe use of drugs. Understanding the structural properties may help pharmacists and chemists enhance treatment outcomes and optimize medication development, leading to more efficient drug creation.

### 7.2 Limitations

The scalability of DTIs is challenging especially when applied to larger datasets. As the size of the drug database grows, the computational complexity of calculating the minimal dominating sets and corresponding topological indices can also increase significantly. This issue could potentially slow down the analysis or require additional computational resources. However, optimization techniques and more efficient algorithms may be developed to address these scalability concerns.

## 8 Conclusion

In this study, we computed domination degree-based topological indices for chemical drugs used in the treatment of heart disease and applied them in QSPR analysis to predict their physicochemical and ADMET properties. The findings reveal that inverse linear, quadratic, and cubic regression models have the potential to predict physicochemical properties, with the cubic regression model demonstrating superior predictive performance for ADMET properties. Multivariate regression models also show potential for predicting physicochemical and ADMET properties. However, the DTIs serve as potential predictors rather than replacements for laboratory experiments. These findings highlight the significance of these indices in establishing a theoretical foundation for drug synthesis, offering a valuable predictive tool for chemists and the pharmaceutical industry. By minimizing the reliance on time-intensive laboratory experiments, these analyses facilitate the efficient design of new drugs by leveraging the strong correlations between critical properties. Additionally, the methodologies presented in this study can be adapted for other drugs targeting different diseases, using computed topological descriptors to predict their respective physicochemical and ADMET properties. Future directions include extending this approach to a broader range of drug classes and exploring the integration of machine learning techniques to further enhance the predictive power of the models. Additionally, further studies may focus on refining the accuracy of predictions for specific ADMET properties by incorporating more complex molecular descriptors.

## Data Availability

The original contributions presented in the study are included in the article/Supplementary Material, further inquiries can be directed to the corresponding author.
