# Supplementary material for: Computation of domination degree-based topological indices using python and QSPR analysis of physicochemical and ADMET properties for heart disease drugs
Source: Front Chem. 2025 Mar 12;13:1536199. doi: 10.3389/fchem.2025.1536199 (PMC11937137; doi:10.3389/fchem.2025.1536199)
Supplement: Supplementary file 1 [file DataSheet1.pdf]

# Supplementary Material

## 1 SUPPLEMENTARY EQUATIONS

$$\phi_d(\Gamma,\alpha,\beta) = \alpha^{840}\beta^{1470} + \alpha^{840}\beta^{1575} + 3\alpha^{1050}\beta^{1386} + \alpha^{1050}\beta^{1428} + \alpha^{1050}\beta^{1050} + 2\alpha^{1050}\beta^{1350} + 2\alpha^{1050}\beta^{1500} + \alpha^{1134}\beta^{1386} + \alpha^{1134}\beta^{1428} + 2\alpha^{1176}\beta^{1260} + \alpha^{1176}\beta^{1386} + 2\alpha^{1350}\beta^{1350} + \alpha^{1386}\beta^{1764} + \alpha^{1386}\beta^{1500} + 2\alpha^{1260}\beta^{1386} + \alpha^{1428}\beta^{1470} + \alpha^{1575}\beta^{1575} + 3\alpha^{1500}\beta^{1650} + \alpha^{1470}\beta^{1680} \tag{S1}$$

$$\frac{1}{MR} = 0.01043 + 17.16386(\frac{1}{DM1^*}) + 168.9257(\frac{1}{DF^*}) - 553.08547(\frac{1}{DM2}) - 417.80007(\frac{1}{DH}) + 331.3677(\frac{1}{DM1}) + 27222.26668(\frac{1}{DF}) \tag{S2}$$

$$\frac{1}{P} = 0.02629 + 43.55058(\frac{1}{DM1^*}) + 501.5468(\frac{1}{DF^*}) - 1433.75061(\frac{1}{DM2}) - 1087.6686(\frac{1}{DH}) + 844.4999(\frac{1}{DM1}) + 6791.5153(\frac{1}{DF}) \tag{S3}$$

$$\frac{1}{MV} = 0.00367 + 2.01472(\frac{1}{DM1^*}) + 529.684(\frac{1}{DF^*}) - 402.6812(\frac{1}{DM2}) - 340.1193(\frac{1}{DH}) + 311.10179(\frac{1}{DM1}) - 2280.89143(\frac{1}{DF}) \tag{S4}$$

$$\frac{1}{MP} = 0.0056 + 27.1.403(\frac{1}{DM1^*}) + 1580.70357(\frac{1}{DF^*}) - 1324.35631(\frac{1}{DM2}) - 654.34931(\frac{1}{DH}) - 73.33825(\frac{1}{DM1}) + 11038.4733(\frac{1}{DF}) \tag{S5}$$

$$\frac{1}{MW} = 0.00268 + 6.51735(\frac{1}{DM1^*}) + 611.0291(\frac{1}{DF^*}) - 349.16936(\frac{1}{DM2}) - 342.83423(\frac{1}{DH}) + 12.89744(\frac{1}{DM1}) + 1540.95395(\frac{1}{DF}) \tag{S6}$$

$$\frac{1}{OC} = 0.6428 - 1138.949(\frac{1}{DM1^*}) + 219346.218(\frac{1}{DF^*}) - 116463.828(\frac{1}{DM2}) + 60975.32915\frac{1}{DH} + 47048.525(\frac{1}{DM1}) - 1121528.252(\frac{1}{DF}) \tag{S7}$$

$$\frac{1}{Caco2} = 2.10382 - 25891.269(\frac{1}{DM1^{*}}) - 3610161.781(\frac{1}{DF^{*}}) + 1282410.474(\frac{1}{DM2}) + 1862119.932(\frac{1}{DH}) + 1266329.075(\frac{1}{DM1}) - 3.30565E7(\frac{1}{DF}) \tag{S8}$$

$$\frac{1}{SKIN} = -0.3734 + 225.0182(\frac{1}{DM1^{*}}) - 32688.52514(\frac{1}{DF^{*}}) + 11856.73865(\frac{1}{DM2}) + 11006.196(\frac{1}{DH}) - 5794.836(\frac{1}{DM1}) + 123415.05436(\frac{1}{DF}) \tag{S9}$$

$$\frac{1}{IA} = 0.01648 - 33.36134(\frac{1}{DM1^{*}}) + 1221.656(\frac{1}{DF^{*}}) + 328.798(\frac{1}{DM2}) + 62.283(\frac{1}{DH}) - 272.578(\frac{1}{DM1}) - 4548.236(\frac{1}{DF}) \tag{S10}$$

$$\frac{1}{TP} = 6.8268 - 78227.9966(\frac{1}{DM1^{*}}) + 6155265.03(\frac{1}{DF^{*}}) - 3671740.470(\frac{1}{DM2}) - 2775764.943(\frac{1}{DH}) + 5077037.241(\frac{1}{DM1}) - 7.66607E7(\frac{1}{DF}) \tag{S11}$$

## 2 SUPPLEMENTARY TABLES

**Table S1.** Actual and predicted values for physicochemical properties using linear, quadratic, and cubic regression models.

| Drugs | 1/MR | | | | 1/P | | | |
|---|---|---|---|---|---|---|---|---|
| | **Actual** | **Linear** | **Quadratic** | **Cubic** | **Actual** | **Linear** | **Quadratic** | **Cubic** |
| Enalapril | 0.01005 | 0.01111 | 0.01139 | 0.01073 | 0.025316 | 0.028 | 0.02873 | 0.02704 |
| Metoprolol | 0.01297 | 0.01248 | 0.01244 | 0.01294 | 0.03268 | 0.03149 | 0.03138 | 0.03265 |
| Propranolol | 0.012658 | 0.01252 | 0.01247 | 0.01299 | 0.031949 | 0.03159 | 0.03146 | 0.03278 |
| Nitroglycerin | 0.02551 | 0.02383 | 0.02467 | 0.02536 | 0.064516 | 0.06018 | 0.06234 | 0.06412 |
| Clopidogrel | 0.011696 | 0.01143 | 0.01162 | 0.01132 | 0.029499 | 0.02881 | 0.02933 | 0.02853 |
| Timolol | 0.012165 | 0.01201 | 0.01207 | 0.01227 | 0.030675 | 0.03029 | 0.03044 | 0.03095 |
| Hydrochlorothiazide | 0.015949 | 0.01675 | 0.0163 | 0.01633 | 0.040161 | 0.04228 | 0.04111 | 0.04119 |
| Furosemide | 0.013193 | 0.01204 | 0.01209 | 0.01232 | 0.033333 | 0.03037 | 0.03051 | 0.03108 |
| Warfarin | 0.011848 | 0.01156 | 0.01172 | 0.01154 | 0.029851 | 0.02914 | 0.02957 | 0.0291 |
| Clonidine | 0.017452 | 0.01681 | 0.01635 | 0.01636 | 0.044053 | 0.04243 | 0.04125 | 0.04127 |
| Atenolol | 0.013459 | 0.01317 | 0.013 | 0.01375 | 0.034014 | 0.03321 | 0.03278 | 0.03473 |
| Sotalol | 0.01387 | 0.01588 | 0.01543 | 0.01585 | 0.034965 | 0.04006 | 0.03892 | 0.04 |
| Bumetanide | 0.010627 | 0.01127 | 0.01151 | 0.01104 | 0.02681 | 0.02842 | 0.02904 | 0.02783 |
| Nadolol | 0.011655 | 0.01168 | 0.01182 | 0.01176 | 0.029412 | 0.02947 | 0.02981 | 0.02965 |
| Esmolol | 0.01224 | 0.01165 | 0.0118 | 0.0117 | 0.030864 | 0.02939 | 0.02975 | 0.02952 |
| Minoxidil | 0.018315 | 0.01739 | 0.01694 | 0.01667 | 0.046296 | 0.04388 | 0.04274 | 0.04205 |
| Methyldopa | 0.018553 | 0.02064 | 0.02058 | 0.01929 | 0.046729 | 0.0521 | 0.05197 | 0.04862 |

**Table S2.** Actual and predicted values for physicochemical properties using linear, quadratic, and cubic regression models.

| Drugs | 1/MP | | | | 1/MW | | | |
|---|---|---|---|---|---|---|---|---|
| | **Actual** | **Linear** | **Quadratic** | **Cubic** | **Actual** | **Linear** | **Quadratic** | **Cubic** |
| Enalapril | 0.006993 | 0.0054 | 0.00772 | 0.00767 | 0.003296 | 0.00333 | 0.00329 | 0.00332 |
| Metoprolol | 0.010227 | 0.00613 | 0.00767 | 0.0077 | 0.003496 | 0.00334 | 0.0033 | 0.00332 |
| Propranolol | 0.008769 | 0.00604 | 0.00772 | 0.0077 | 0.00329 | 0.00332 | 0.00328 | 0.00332 |
| Nitroglycerin | 0.013333 | 0.0058 | 0.00762 | 0.00758 | 0.004827 | 0.00336 | 0.00342 | 0.00339 |
| Clopidogrel | 0.010853 | 0.00556 | 0.00768 | 0.00762 | 0.003029 | 0.00319 | 0.0031 | 0.00309 |
| Timolol | 0.013986 | 0.00556 | 0.00762 | 0.00756 | 0.00316 | 0.00329 | 0.00326 | 0.00326 |
| Hydrochlorothiazide | 0.003663 | 0.00965 | 0.00522 | 0.00552 | 0.003359 | 0.00395 | 0.0042 | 0.0042 |
| Furosemide | 0.004854 | 0.00557 | 0.00761 | 0.00755 | 0.003024 | 0.00329 | 0.00327 | 0.00327 |
| Warfarin | 0.006211 | 0.00546 | 0.00769 | 0.00761 | 0.003244 | 0.00323 | 0.00313 | 0.00313 |
| Clonidine | 0.007692 | 0.00905 | 0.00552 | 0.00582 | 0.004346 | 0.00396 | 0.00421 | 0.00421 |
| Atenolol | 0.006849 | 0.00604 | 0.00729 | 0.00732 | 0.003755 | 0.00345 | 0.00354 | 0.00357 |
| Sotalol | 0.004843 | 0.00844 | 0.00584 | 0.00612 | 0.003671 | 0.00383 | 0.00407 | 0.00409 |
| bumetanide | 0.004348 | 0.00541 | 0.00772 | 0.00763 | 0.002744 | 0.00319 | 0.00306 | 0.00304 |
| Nadolol | 0.008065 | 0.00548 | 0.00767 | 0.0076 | 0.003232 | 0.00324 | 0.00317 | 0.00317 |
| Esmolol - | - | 0.00546 | 0.00768 | 0.00761 | 0.003386 | 0.00324 | 0.00316 | 0.00316 |
| Minoxidil - | - | 0.01003 | 0.00504 | 0.00534 | 0.004779 | 0.00403 | 0.00428 | 0.00427 |
| Methyldopa | 0.003333 | 0.01535 | 0.00331 | 0.00297 | 0.004735 | 0.00449 | 0.00452 | 0.00446 |

**Table S3.** Actual and predicted values for physicochemical properties using linear, quadratic, and cubic regression models.

| Drugs | 1/MV | | | |
|---|---|---|---|---|
| | Actual | Linear | Quadratic | Cubic |
| Enalapril | 0.003199 | 0.00431 | 0.00405 | 0.00391 |
| Metoprolol | 0.003865 | 0.00432 | 0.00409 | 0.00398 |
| Propranolol | 0.004216 | 0.00432 | 0.00409 | 0.00399 |
| Nitroglycerin | 0.007358 | 0.00818 | 0.00703 | 0.00735 |
| Clopidogrel | 0.004093 | 0.00431 | 0.00405 | 0.00392 |
| Timolol | 0.003868 | 0.00431 | 0.00406 | 0.00394 |
| Hydrochlorothiazide | 0.005688 | 0.00502 | 0.00585 | 0.00643 |
| Furosemide | 0.004859 | 0.00432 | 0.00407 | 0.00395 |
| Warfarin | 0.004241 | 0.00431 | 0.00405 | 0.00392 |
| Clonidine | 0.006527 | 0.00485 | 0.00546 | 0.00601 |
| Atenolol | 0.004225 | 0.00435 | 0.00416 | 0.00411 |
| Sotalol | 0.004552 | 0.00463 | 0.00493 | 0.00533 |
| Bumetanide | 0.003697 | 0.00431 | 0.00405 | 0.00391 |
| Nadolol | 0.003846 | 0.00431 | 0.00405 | 0.00392 |
| Esmolol | 0.003671 | 0.00431 | 0.00405 | 0.00392 |
| Minoxidil | 0.007267 | 0.00508 | 0.00597 | 0.00654 |
| Methyldopa | 0.006645 | 0.00657 | 0.00781 | 0.00669 |

**Table S4.** Actual and predicted values for physicochemical properties using multilinear regression models.

| Drugs | 1/MR | | 1/P | | 1/MV | | 1/MP | | 1/MW | |
|---|---|---|---|---|---|---|---|---|---|---|
| | **Actual** | **Predicted** | **Actual** | **Predicted** | **Actual** | **Predicted** | **Actual** | **Predicted** | **Actual** | **Predicted** |
| Enalapril | 0.01005 | 0.01064 | 0.025316 | 0.0268 | 0.003199 | 0.00363 | 0.006993 | 0.00575 | 0.002657 | 0.00269 |
| Metoprolol | 0.01297 | 0.01312 | 0.03268 | 0.03312 | 0.003865 | 0.0043 | 0.008333 | 0.0089 | 0.00374 | 0.00366 |
| Propranolol | 0.012658 | 0.01309 | 0.031949 | 0.03305 | 0.004216 | 0.00427 | 0.010417 | 0.00885 | 0.003856 | 0.00366 |
| Nitroglycerin | 0.02551 | 0.02551 | 0.064516 | 0.06452 | 0.007358 | 0.00736 | 0.074074 | 0.07408 | 0.004404 | 0.0044 |
| Clopidogrel | 0.011696 | 0.01131 | 0.029499 | 0.02851 | 0.004093 | 0.00378 | 0.006329 | 0.00681 | 0.003108 | 0.00298 |
| Timolol | 0.012165 | 0.0123 | 0.030675 | 0.03105 | 0.003868 | 0.00403 | 0.013986 | 0.00812 | 0.00316 | 0.00338 |
| Hydrochlorothiazide | 0.015949 | 0.01637 | 0.040161 | 0.04128 | 0.005688 | 0.00619 | 0.003663 | 0.00428 | 0.003359 | 0.0039 |
| Furosemide | 0.013193 | 0.0124 | 0.033333 | 0.03129 | 0.004859 | 0.00408 | 0.004854 | 0.00816 | 0.003024 | 0.0034 |
| Warfarin | 0.011848 | 0.01154 | 0.029851 | 0.0291 | 0.004241 | 0.00384 | 0.006211 | 0.00712 | 0.003244 | 0.00307 |
| Clonidine | 0.017452 | 0.01778 | 0.044053 | 0.0449 | 0.006527 | 0.00664 | 0.007692 | 0.00711 | 0.004346 | 0.00445 |
| Atenolol | 0.013459 | 0.01368 | 0.034014 | 0.03453 | 0.004225 | 0.00438 | 0.006849 | 0.00923 | 0.003755 | 0.00388 |
| Sotalol | 0.01387 | 0.01377 | 0.034965 | 0.0347 | 0.004552 | 0.00445 | 0.004843 | 0.00451 | 0.003671 | 0.00359 |
| Bumetanide | 0.010627 | 0.01099 | 0.02681 | 0.0277 | 0.003697 | 0.00371 | 0.004348 | 0.00632 | 0.002744 | 0.00284 |
| Nadolol | 0.011655 | 0.01177 | 0.029412 | 0.02969 | 0.003846 | 0.00389 | 0.008065 | 0.00747 | 0.003232 | 0.00317 |
| Esmolol | 0.01224 | 0.01173 | 0.030864 | 0.02958 | 0.003671 | 0.00387 | - | 0.00745 | 0.003386 | 0.00316 |
| Minoxidil | 0.018315 | 0.01763 | 0.046296 | 0.0445 | 0.007267 | 0.00674 | - | 0.00517 | 0.004779 | 0.00424 |
| Methyldopa | 0.018553 | 0.01859 | 0.046729 | 0.04681 | 0.006645 | 0.00665 | 0.003333 | 0.00329 | 0.004735 | 0.00473 |
